# Supplementary figures and images for: STK19 positions TFIIH for cell-free transcription-coupled DNA repair
Source: Cell. Author manuscript; Available in PMC 2024 Dec 14. (PMC11645862; doi:10.1016/j.cell.2024.10.020)

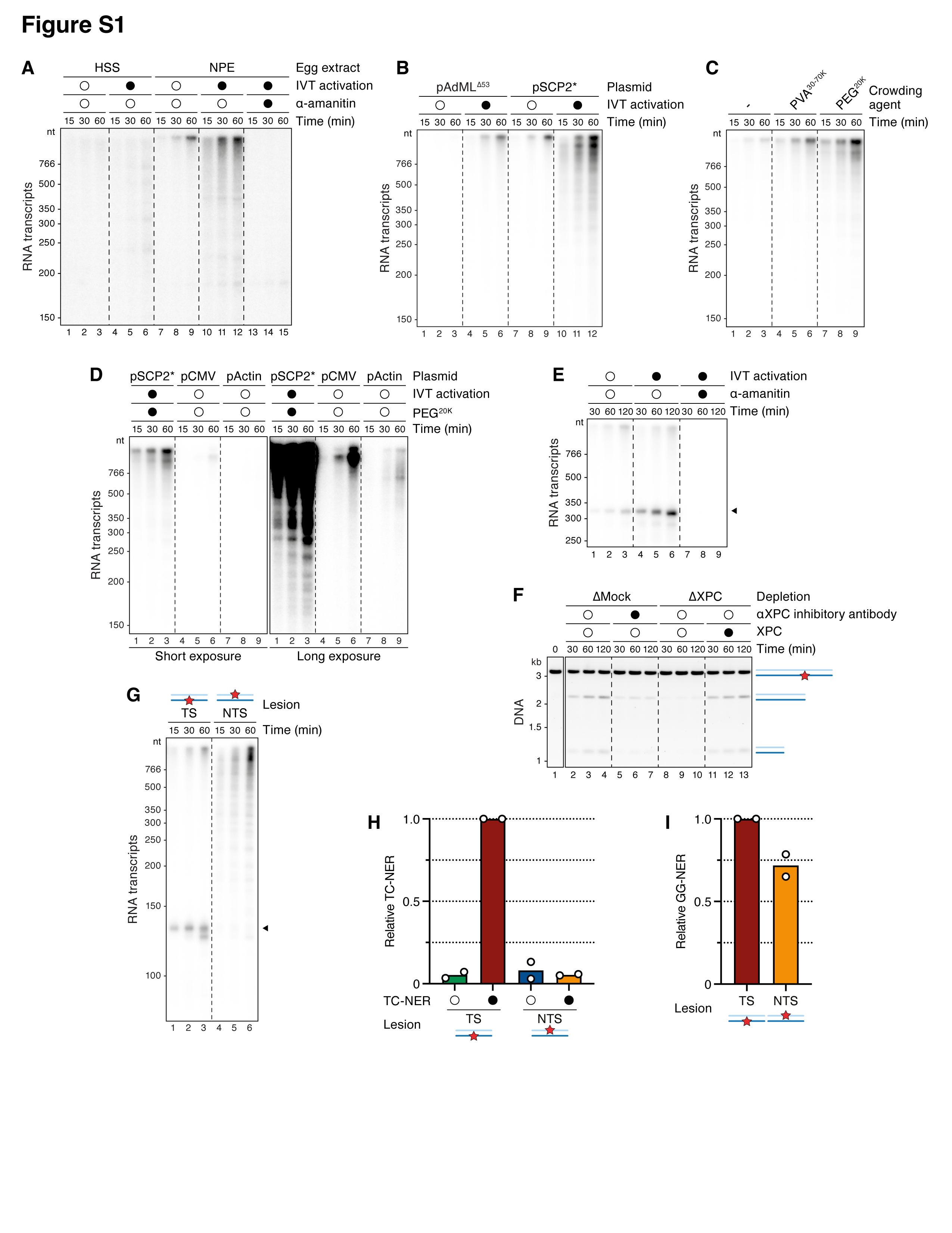

Supplement: 1 — Figure S1. Characterization and optimization of in vitro transcription, GG-NER, and TC-NER in frog egg extracts, related to Figure 1 (A) Plasmid pAdMLΔ53 (adenovirus major late promoter flanked by UAS sites) was incubated with total egg lysate (HSS, high speed supernatant) or NPE containing [α-32P]UTP that were also optionally supplemented with the transcription activator GAL4-VP64 and TBP (for in vitro transcription (IVT) activation) and 2 μM α-amanitin. At the indicated times, RNA was recovered, separated on a Urea-PAGE gel, and subjected to autoradiography. Open and closed circles indicate the absence or presence of a given condition, respectively. (B) Cell-free transcription was carried out in NPE as in (A), comparing plasmids containing the adenovirus major late promoter and a modified super core promoter 2, SCP2* (see Methods). (C) Cell-free transcription was carried out in NPE as in (A), with the indicated crowding agents being added to a final concentration of 1% (v/v). (D) Comparison of our optimal inducible transcription condition (pSCP2* substrate, IVT activation, and 1% PEG20K) to transcription from the CMV and endogenous actin promoters. Plasmids pCMV and pActin were added to NPE without any supplements as in 34. Short and long exposures of the autoradiograph are shown. (E) The reactions described in Figure 1C were supplemented with [α-32P]UTP and used to monitor transcription, demonstrating that IVT activation strongly stimulated transcription, and that α-amanitin inhibited transcription in this experiment. (F) NPE supports GG-NER. Plasmid pAdMLΔ53 containing a cisplatin 1,3-GTG intrastrand crosslink was incubated in mock-depleted NPE supplemented with buffer or inhibitory XPC antibody, or in NPE depleted of XPC that was optionally supplemented with XPC protein purified from egg extract. DNA was recovered, and PmlI site regeneration was monitored (as depicted in Figure 1A). DNA was separated by agarose gel electrophoresis and visualized using SYBR Gold [file NIHMS2030424-supplement-1.tif]

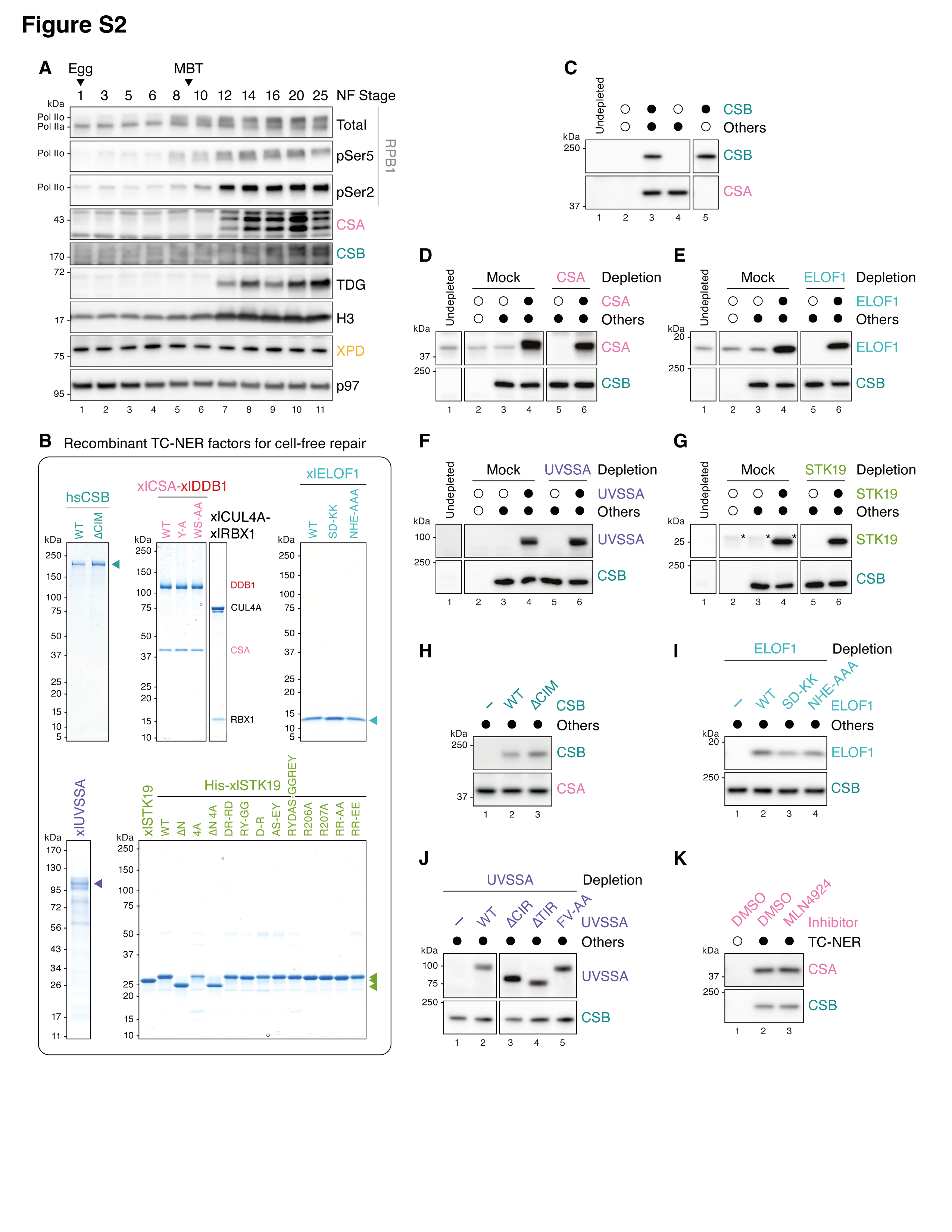

Supplement: 2 — Figure S2. Characterization and purification of TC-NER factors, related to Figure 2 (A) Frog eggs were fertilized in vitro, and at the indicated developmental stages (NF, Nieuwkoop and Faber46), embryo extracts47 were blotted for the indicated proteins. Transcription activation during the mid-blastula transition (MBT) was detected by hyperphosphorylation of the RPB1 subunit of Pol II. Protein levels of the TC-NER factors CSA and CSB followed a similar trend as the developmentally regulated TDG (Thymine DNA Glycosylase),47 being only detectable after the MBT. In contrast, p97 and the TFIIH subunit XPD were similarly abundant at all stages. (B) Proteins used for cell-free TC-NER assays throughout the paper were separated by SDS-PAGE and stained with Coomassie blue. H. sapiens CSB was used throughout our study because it activated TC-NER as efficiently as X. laevis CSB (not shown) while exhibiting slightly better protein stability. For all other TC-NER factors, X. laevis proteins were used. (C) Representative western blot of the extracts used for conditions III and IV in Figure 2A. Each error-free repair assay included reactions in the absence (lane 2) and presence (lane 3) of all TC-NER factors, corresponding to conditions I and II in Figure 2A. The CSA blot is representative of the “Other” TC-NER factors, other than CSB. Note that CSB is absent in NPE and therefore not detected in undepleted egg extract (lane 1). (D-G) Representative western blots of the extracts used in Figure 2A (conditions V-VIII) and Figure 2B (conditions III-X). Each error-free repair assay included reactions in the absence (lane 2) and presence (lane 4) of all TC-NER factors, corresponding to conditions I and II in both Figures 2A and 2B. In each panel, lane 3 shows the extract used for conditions V-VIII in Figure 2A, respectively. Lanes 5 and 6 correspond to the depletion and add-back samples for CSA (D), ELOF1 (E), UVSSA (F), and STK19 (G), respectively, shown in conditions III-X in Figure 2B [file NIHMS2030424-supplement-2.tif]

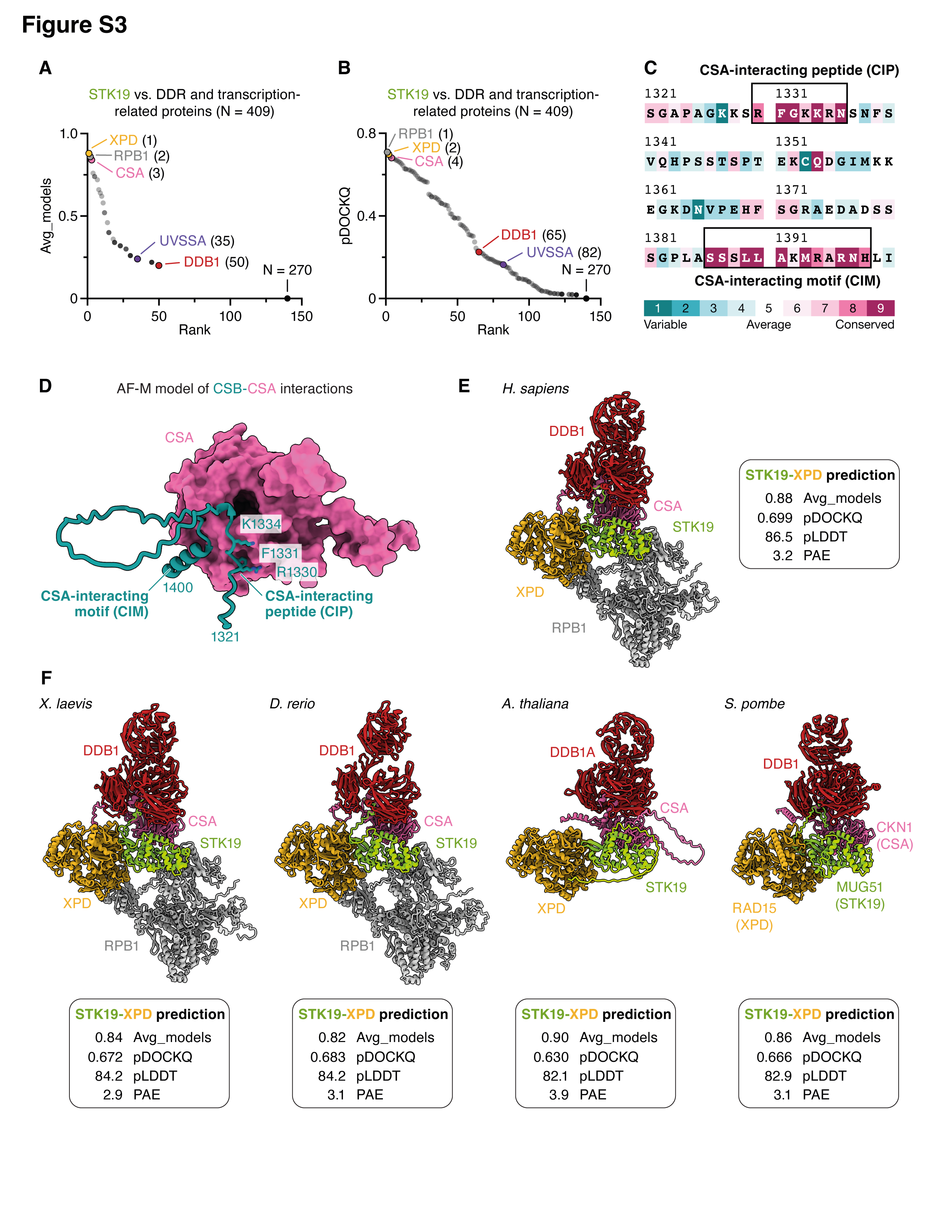

Supplement: 3 — Figures S3: AlphaFold-Multimer structure prediction results, related to Figure 3 (A-B) The ~400 binary structure predictions shown in Figure 3A were ranked based on avg_models (A), a confidence metric that quantifies the agreement among the five different AF-M models, or pDOCKQ (B). (C) Sequence conservation of human CSB residues 1321–1400 calculated in ConSurf.48 The CSA-interacting peptide (CIP) and the CSA-interacting motif (CIM) of CSB are highly conserved, whereas residues flanking these regions are more variable. (D) AF-M prediction for the interaction between CSB and CSA within the folded TC-NER complex (Figures 3B and 3C). CSA is shown in surface representation, and CSB residues 1321–1400 are depicted in cartoon representation. Key residues of CSB in the CSA-interacting peptide (CIP) identified here are shown as side chains. (E-F) Composite structure prediction of STK19, DDB1, CSA, XPD, and RPB1 complex in human (E) and other organisms (F). STK19 was folded separately with CSA-DDB1, RPB1, or XPD, and the resulting models were aligned on STK19 to show a composite complex. No interaction was predicted for STK19 with RPB1 in A. thaliana and S. pombe. The confidence metrics shown refer to the binary STK19-XPD prediction. [file NIHMS2030424-supplement-3.tif]

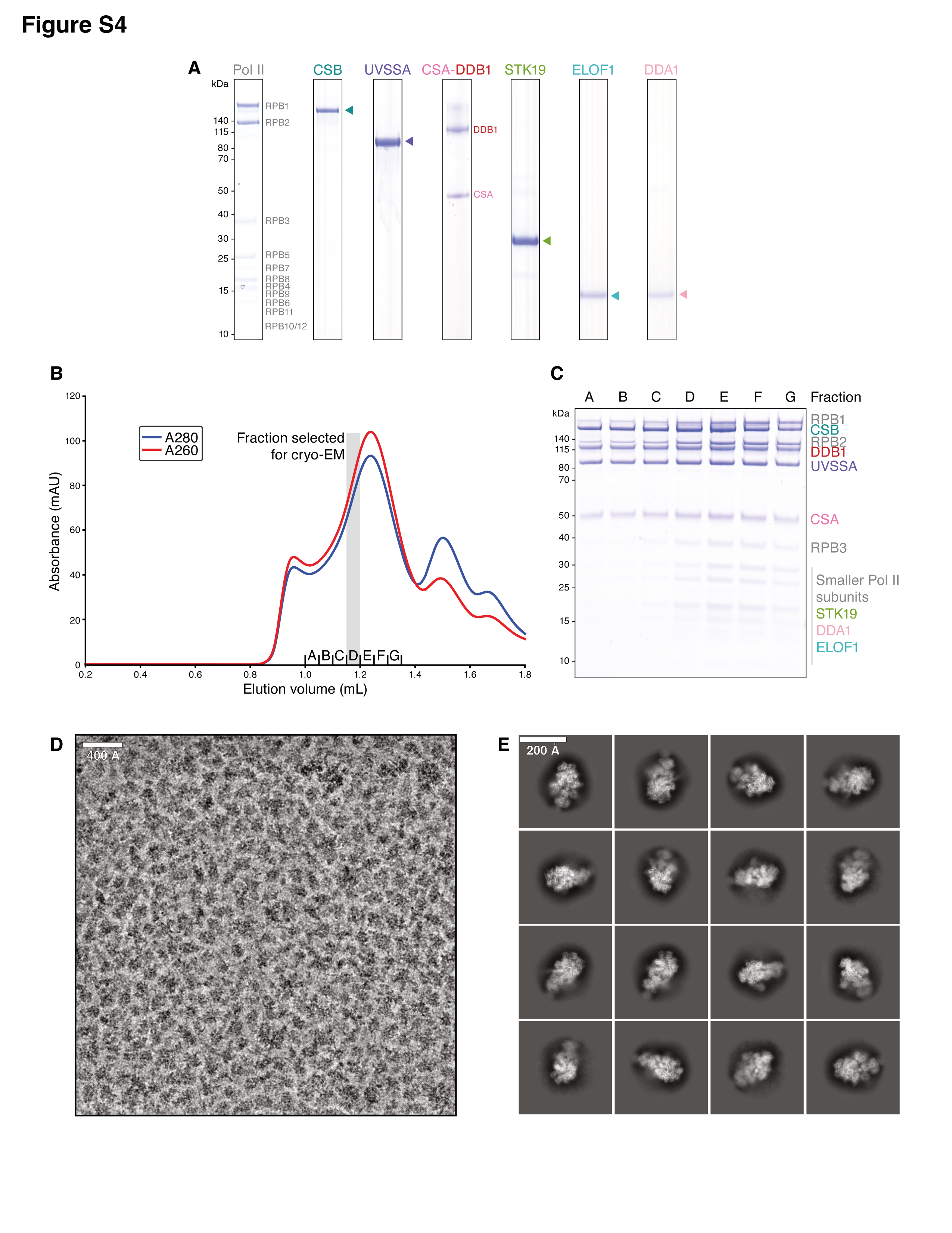

Supplement: 4 — Figure S4: Complex formation and cryo-EM data analysis, related to Figure 3 (A) SDS-PAGE of purified S. scrofa RNA polymerase II and H. sapiens TC-NER factors, including STK19 and DDA1. (B) Chromatogram of TC-NER complex formation via size-exclusion chromatography. (C) SDS-PAGE of fractions from (B). (D) Representative micrograph from cryo-EM data collection. Scale bar, 400 Å. (E) 2D classes of TC-NER complex. Scale bar, 200 Å. [file NIHMS2030424-supplement-4.tif]

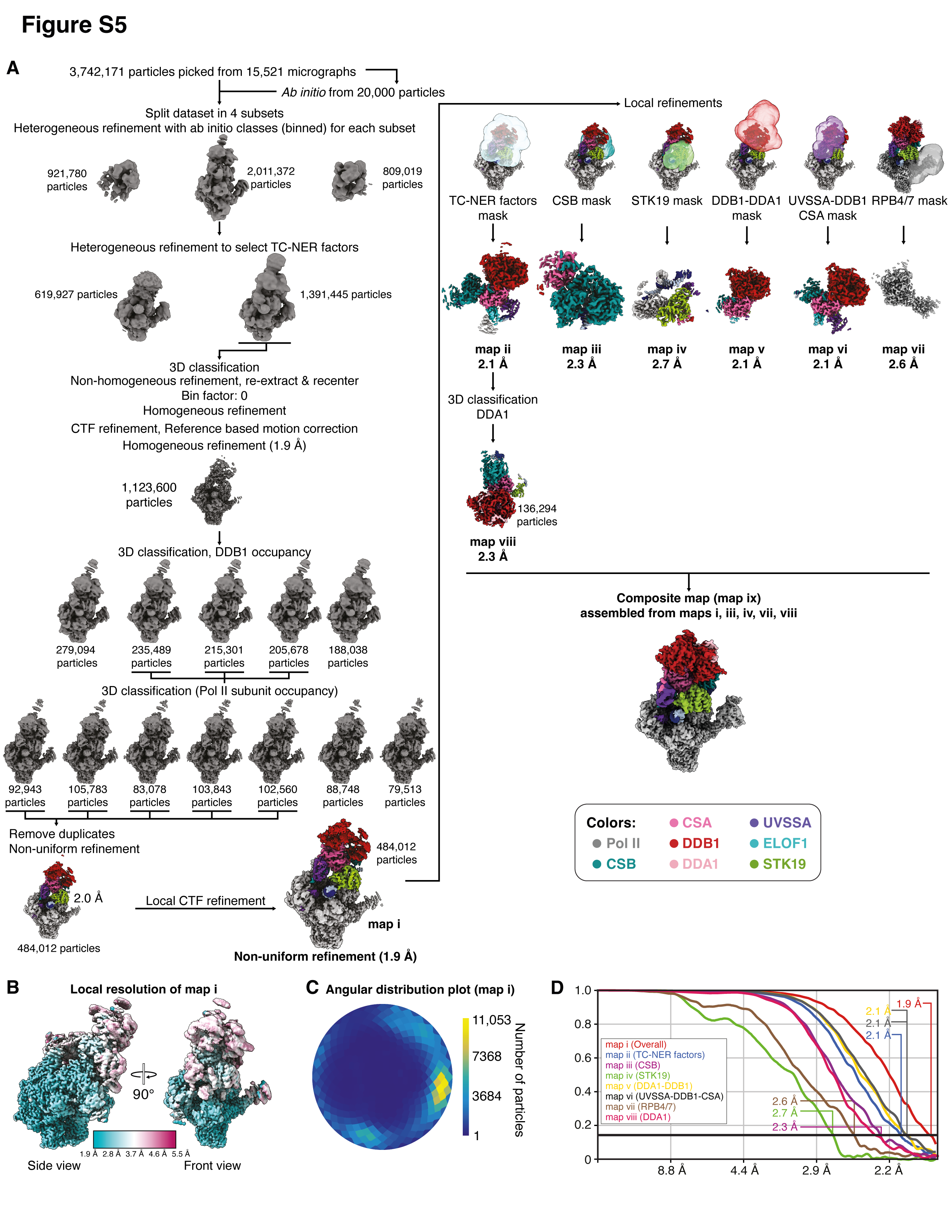

Supplement: 5 — Figure S5: Classification tree and cryo-EM data analysis metrics, related to Figure 3 (A) Classification tree of cryo-EM data analysis. Particle numbers and resolutions are indicated. (B) Local resolution as colored on map i. (C) Angular distribution plot of particle assignment (map i). (D) Fourier shell correlation (FSC) curves of maps i-viii. FSC 0.143 criterion and achieved resolutions are indicated. [file NIHMS2030424-supplement-5.tif]

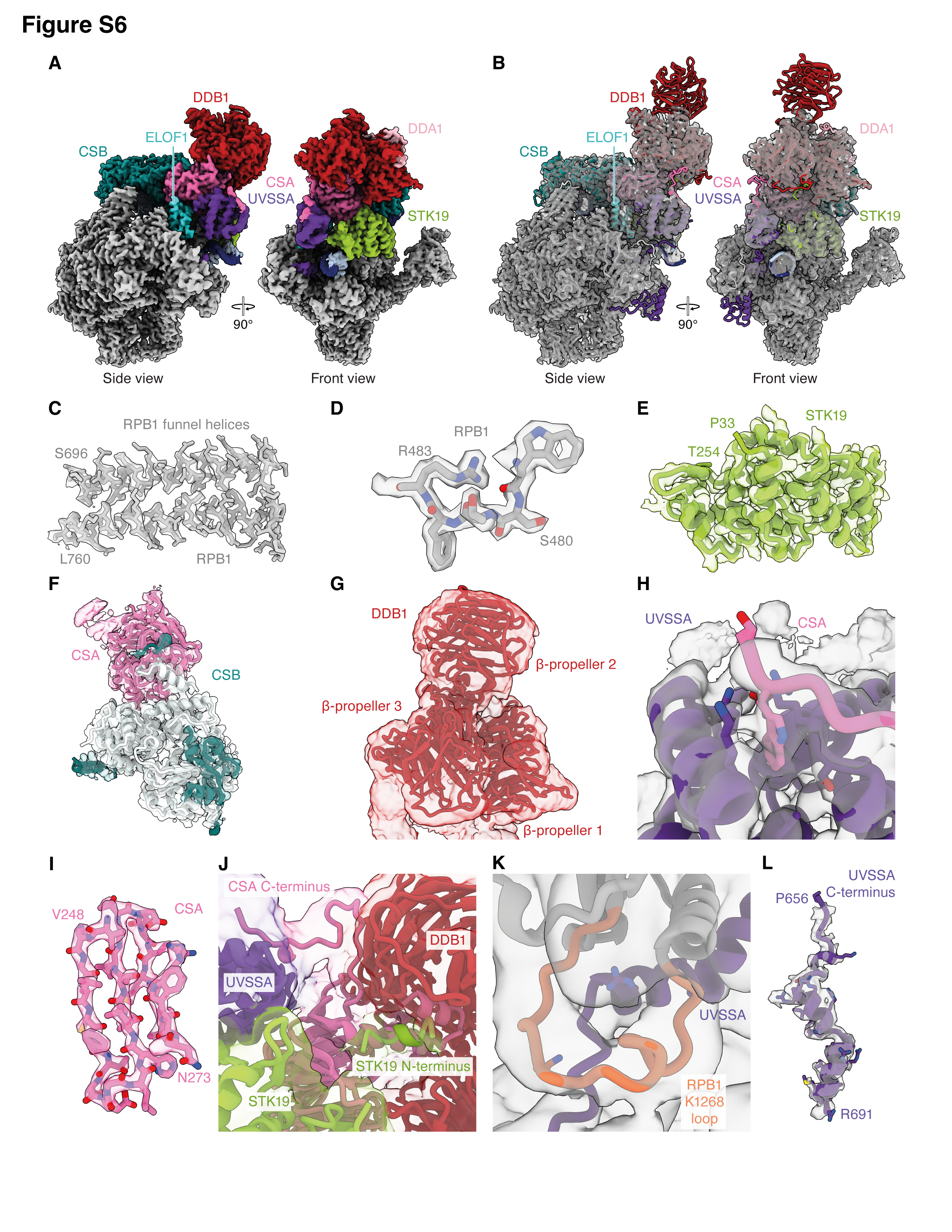

Supplement: 6 — Figure S6: Cryo-EM data quality, related to Figure 3 (A) Coulomb potential map of RNA polymerase II TC-NER complex (composite map ix). (B) Coulomb potential map of RNA polymerase II TC-NER complex (grey, composite map ix) with fitted model. (C) RPB1 funnel helices with corresponding density (map i, sharpened). (D) Features of RPB1 resolves aromatic rings (map i, sharpened). (E) Coulomb potential map with corresponding model of STK19 (map iv, DeepEMhanced). (F) Coulomb potential map with corresponding model of CSA and CSB (map iii, DeepEMhanced). (G) Coulomb potential map with corresponding model of DDB1 (map ii, low-pass filtered). (H) Coulomb potential map with corresponding model of UVSSA and CSA C-terminus (map i, low-pass filtered). (I) Coulomb potential map with corresponding model of CSA residues 248–273 (map ii, sharpened. (J) Coulomb potential map with corresponding model of CSA C-terminus, STK19 Nterminus, UVSSA and DDB1 248–273 (map i, low-pass filtered). (K) Coulomb potential map with corresponding model of RPB1 K1268 loop and UVSSA C-terminus (map i, low-pass filtered). (L) Coulomb potential map with corresponding model of UVSSA C-terminus (map i). [file NIHMS2030424-supplement-6.tif]

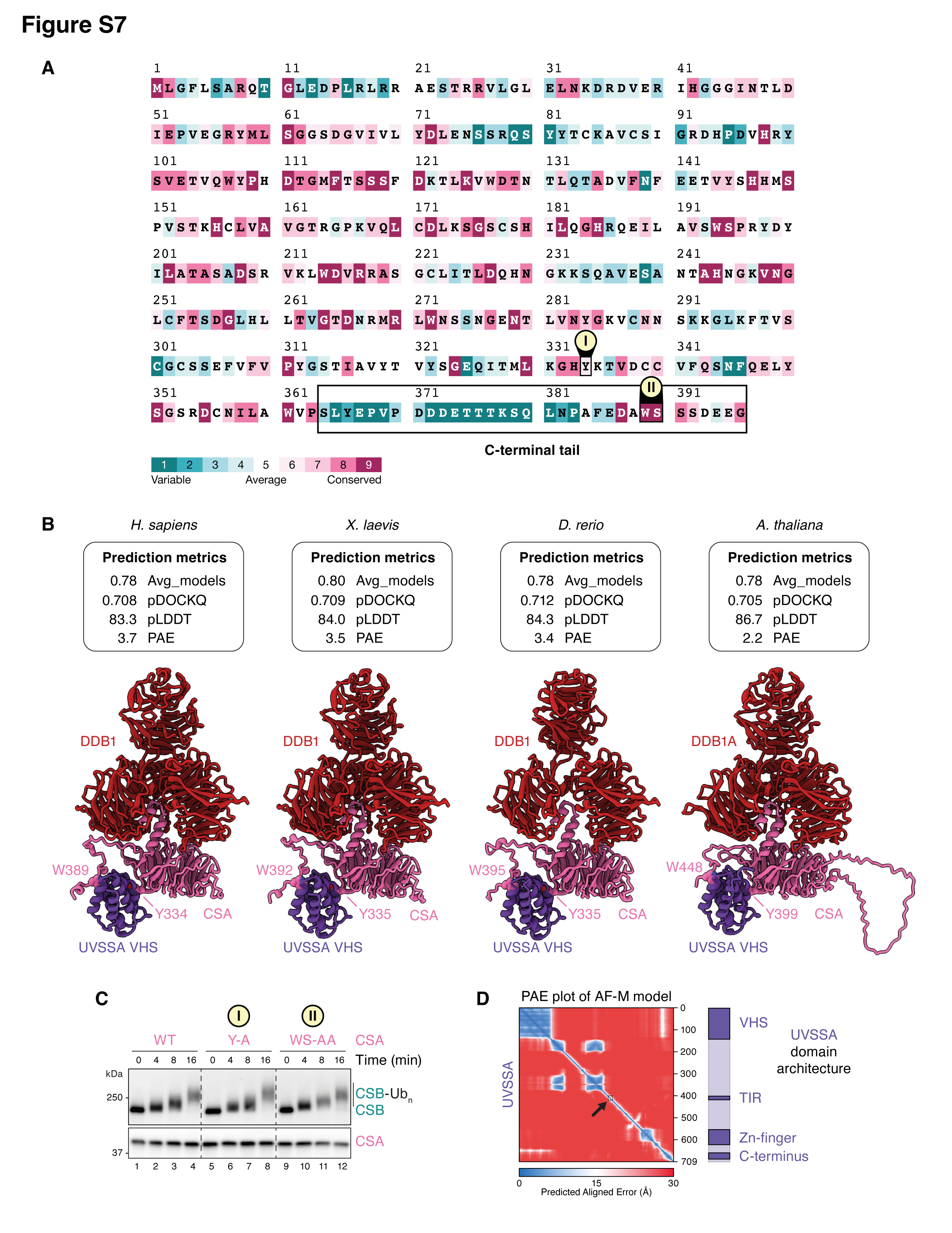

Supplement: 7 — Figure S7: The interaction of the C-terminal tail of CSA with UVSSA is conserved, related to Figure 6 (A) Sequence conservation of human CSA calculated in ConSurf.48 CSA residues involved in the interaction with the UVSSA VHS domain as described in Figure 6A and 6B are highlighted. (B) AF-M structure prediction of full-length UVSSA in complex with CSA-DDB1 in the indicated organisms. For simplicity, only the VHS domain of UVSSA bound by CSA is depicted. Side chains of CSA residues interacting with UVSSA are shown. Confidence metrics quantify the interaction between UVSSA and the CSA-DDB1 heterodimer. (C) In vitro ubiquitination assay like the one shown in Figure 6C, except that UVSSA was replaced by human CSB and UBE2E1 was replaced by UBE2D2. Reaction products were immunoblotted for CSA and CSB. CSB-Ubn, polyubiquitinated CSB. (D) PAE plot for the predicted full-length structure of human UVSSA and its domain architecture. The location of the TFIIH-interacting region (TIR) within the PAE plot is highlighted by an arrow. [file NIHMS2030424-supplement-7.tif]
