# Supplementary material for: STK19 positions TFIIH for cell-free transcription-coupled DNA repair
Source: Cell. Author manuscript; Available in PMC 2024 Dec 14. (PMC11645862; doi:10.1016/j.cell.2024.10.020)
Supplement: 8 [file NIHMS2030424-supplement-8.pdf]

Table S2: Cryo-EM data collection, refinement and validation statistics, related to Figure 3

	**Map i (Pol II TC-NER complex)** PDB 9BZ0 EMD-45050 EMD-47273	**Map ii (TC-NER factors)** EMD-47263	**Map iii (CSB)** EMD-47262	**Map iv (STK19)** EMD-47261	**Map v (DDB1-DDA1)** EMD-47266	**Map vi (UVSSA-DDB1-CSA)** EMD-47267	**Map vii (RPB4/7)** EMD-47271	**Map viii (DDA1)** EMD-47272
**Data collection and processing**								
Magnification	130,000	130,000	130,000	130,000	130,000	130,000	130,000	130,000
Voltage (kV)	300	300	300	300	300	300	300	300
Electron exposure ($e^{-}/Å^2$)	52.4	52.4	52.4	52.4	52.4	52.4	52.4	52.4
Defocus range (μm)	0.6-1.8	0.6-1.8	0.6-1.8	0.6-1.8	0.6-1.8	0.6-1.8	0.6-1.8	0.6-1.8
Pixel size (Å)	0.94	0.94	0.94	0.94	0.94	0.94	0.94	0.94
Symmetry imposed	C1	C1	C1	C1	C1	C1	C1	C1
Initial particle images (no.)	3,742,171	3,742,171	3,742,171	3,742,171	3,742,171	3,742,171	3,742,171	3,742,171
Final particle images (no.)	484,012	484,012	484,012	484,012	484,012	484,012	484,012	136,294
Map resolution (Å)<br>FSC threshold	1.9<br>0.143	2.1<br>0.143	2.3<br>0.143	2.7<br>0.143	2.1<br>0.143	2.1<br>0.143	2.6<br>0.143	2.3<br>0.143
Map resolution range (Å)	1.9-5.5							
**Refinement**								
Initial models used (PDB ID)	8B3D, AF-M predictions							
Map sharpening *B* factor ($Å^2$)	-38.3	-43.2	-58	-54.0	-46.2	-47.6	-58.3	-40.8
Model composition<br>Non-hydrogen atoms<br>Protein residues<br>Nucleotides<br>Ligands	 54,682<br>6636<br>94<br>12							
B factors ($Å^2$)<br>Protein<br>Nucleotide<br>Ligand	 52.85<br>63.08<br>74.30							
R.m.s. deviations<br>Bond lengths (Å)<br>Bond angles (°)	 0.009<br>0.865							
**Validation**<br>MolProbity score<br>Clashscore<br>Poor rotamers (%)	 1.30<br>2.7<br>0.28							
Ramachandran plot<br>Favored (%)<br>Allowed (%)<br>Disallowed (%)	 96.36<br>3.62<br>0.02							
CC box (Model vs. Data)	0.81							

Table S3: Input structural models and model confidence, related to Figure 3

Subunit	Chain id(s)	Input model	Level of confidence
RPB1	A	8B3D	Atomic, RPB1 K1268 loop: secondary structure confidence
RPB2	B	8B3D	Atomic
RPB3	C	8B3D	Atomic
RPB4	D	8B3D	Secondary structure
RPB5	E	8B3D	Atomic
RPB6	G	8B3D	Atomic
RPB7	F	8B3D	Secondary structure
RPB8	H	8B3D	Atomic
RPB9	I	8B3D	Atomic
RPB10	J	8B3D	Atomic
RPB11	K	8B3D	Atomic
RPB12	L	8B3D	Atomic
ELOF1	M	8B3D	Atomic
Template DNA	T	8B3D	Atomic
Non-template DNA	N	8B3D	Atomic
RNA	P	8B3D	Atomic
ERCC8/CSA	a	8B3D	Atomic
ERCC6/CSB	b	AlphaFold2	Atomic
UVSSA	c	8B3D	Atomic, UVSSA Zn finger: clipped and rigid body docked, UVSSA C-terminus: clipped
DDB1	d	AlphaFold-Multimer of DDB1/DDA1 model	Atomic, β-propeller 2: rigid body docked
DDA1	e	AlphaFold-Multimer of DDB1/DDA1 model	Atomic
STK19	f	AlphaFold-Multimer, *De novo*	Atomic, STK19 N-terminus: Backbone level confidence, no register confidence
